# Regeneration of the Gastrointestinal Tract After Using a Small Intestine Submucosa Patch—A Rat Model

**DOI:** 10.3390/biomedicines13102397

**Published:** 2025-09-30

**Authors:** Tamas Toth, Radu-Alexandru Prisca, Emoke Andrea Szasz, Reka Borka-Balas, Angela Borda

**Affiliations:** 1 Institution Organizing University Doctoral Studies (IOSUD), Pediatric Surgery and Orthopedics Department, George Emil Palade University of Medicine, Pharmacy, Science and Technology of Targu Mures, 540139 Targu Mures, Romania; tamas.toth@umfst.ro; 2 Pediatric Surgery and Orthopedics Department, Targu Mures Emergency Clinical County Hospital, George Emil Palade University of Medicine, Pharmacy, Science and Technology of Targu Mures, 540139 Targu Mures, Romania; 3 Histology Department, George Emil Palade University of Medicine, Pharmacy, Science and Technology of Targu Mures, 540139 Targu Mures, Romania; 4 Pediatric Clinic I, Targu Mures Emergency Clinical County Hospital, George Emil Palade University of Medicine, Pharmacy, Science and Technology of Targu Mures, 540139 Targu Mures, Romania

**Keywords:** small intestine submucosa, extracellular matrix, gastrointestinal perforation, stomach defect, small intestinal defect, large intestine defect, gastrointestinal tract regeneration, regenerative medicine

## Abstract

**Background:** Necrotizing enterocolitis (NEC) is a life-threatening condition characterized by necrosis of the gastrointestinal tract caused by hypoperfusion and hypoxia-induced inflammation. Surgical treatment often requires resection, with high morbidity and mortality. Intestinal tissue engineering using absorbable biomaterials represents a potential alternative. Small intestinal submucosa (SIS) is a biodegradable extracellular matrix (ECM) scaffold that may facilitate regeneration of the native tissue. **Objectives:** The aim of our study is to investigate the regenerative potential of SIS in a rat model with multiple gastrointestinal defects. **Methods:** In rats, after a midline laparotomy, an approximately 1 cm full-thickness incision was performed on the anterior gastric wall, on the antimesenteric side of the small and large intestine, each covered with an SIS patch. After three weeks, the graft sites and adjacent fragments were harvested and fixed in 10% neutral buffered formalin. Cross-sections of the grafted area were processed and stained with hematoxylin and eosin for histologic analysis. **Results:** Among the fifteen Wistar rats used in the study, the survival rate was 80% (12/15). Macroscopic examination of the abdominal cavity after the second surgery showed no complications. Adhesions were present in 92% (11/12). Histological examination demonstrated complete mucosal coverage in all stomach samples, nine of the small intestine, and ten of the large intestine. Mild fibrosis with minimal inflammatory infiltrates predominated. Ulceration with granulation tissue replacement was observed in three small intestine samples. Foreign body reactions were restricted to suture sites. **Conclusions:** In this multifocal injury model, SIS integrated effectively and supported early regenerative healing across gastric, small-intestinal, and colonic sites at 3 weeks. These data support further studies with longer follow-up, quantitative histology and functional assessment, and evaluation in neonatal-relevant large animal models to determine translational potential for NEC surgery.

## 1. Introduction

Necrotizing enterocolitis (NEC) is a devastating clinical condition in preterm infants with long-term complications in surgical cases, such as neurodevelopmental impairment, short bowel syndrome, and intestinal stricture [1]. It is characterized by local or diffuse necrosis of the gastrointestinal tract caused by hypoperfusion and hypoxia-induced inflammation [2]. The most frequently affected segments are the colon and the distal ileum. The mortality rate in NEC ranges from 15% to 30% [3].

Current treatment strategies are limited, and when surgical intervention is required, they often involve bowel resection. Surgical approach is selected in case of pneumoperitoneum or when the clinical condition deteriorates despite maximal medical treatment. Surgical intervention, in the absence of intestinal perforation or full-thickness necrosis of the intestinal wall, could reduce mortality and complication rate [4].

Surgical treatment has not changed over time. It includes peritoneal drainage, laparotomy with resection of the necrotic intestine, ostomy creation, or end-to-end anastomosis between the unaffected segments. Sufficient blood supply to the ends being anastomosed, minimal tension between edges, adequate preoperative preparation, and proper surgical technique are recommended to prevent anastomotic dehiscence.

The outcomes of extensive resections underline the need for new therapeutic approaches. Thus, strategies that promote length preservation and regeneration of the gastrointestinal tract are particularly important.

Tissue-engineering approaches using biodegradable scaffolds have emerged as promising alternatives. Small intestinal submucosa (SIS) is a biodegradable, commercially available, acellular, and immunologically inert collagen-based matrix, primarily composed of fibrillar collagen. SIS aids in the regeneration of the native tissue by providing support for the normal architecture of mucosa, muscularis, and serosa. It is successfully used in experimental studies as an adjunct to repair blood vessels, tendons, or as bladder or ureter grafts [5,6,7,8]. In the regeneration of the digestive tube, some experimental studies evaluated SIS on the esophagus, stomach, small bowel, descending colon, and biliary duct defects [9,10,11,12]. Clinical experience with SIS is limited. It was used in vaginal reconstruction, inguinal and incisional hernia repair, pediatric bladder augmentation, and aortic arch reparation [13,14,15,16,17]. The inconsistent results and the number of reduced clinical studies demonstrate the need for further studies. The Food and Drug Administration (FDA) approved the use of SIS to reinforce soft tissues or abdominal wall defects and wound dressings using SIS. Its role in addressing complex gastrointestinal injuries involving multiple segments remains undetermined.

In the present experimental animal study, we aimed to investigate the regenerative potential of SIS patches in a rat model with multiple full-thickness gastrointestinal defects, focusing on macroscopic and histological outcomes. This design simulates the multifocal injury often seen in NEC and allows a broader assessment of regenerative outcomes across different tissue environments. To our knowledge, this is the first study to assess SIS patch integration simultaneously in these three gastrointestinal segments in a rat model. Demonstrating effective regeneration across different segments would justify further histomorphometrical and biomechanical studies prior to translational studies.

## 2. Materials and Methods

### 2.1. Study Site and Ethical Approvals

The experimental study was conducted at the Scientific Research and Technological Development Unit of “George Emil Palade” University of Medicine, Pharmacy, Sciences and Technology of Târgu Mureş, an accredited facility operating under continuous veterinary supervision.

The protocol was designed in accordance with the ARRIVE 2.0 guidelines and was approved by the Scientific Research Ethics Committee of the same institution (approval no. 3347/13.09.2024) and by the National Sanitary Veterinary and Food Safety Authority (approval no. 73/03.12.2024). All procedures followed Law No. 43/2014 of the Romanian Parliament and Directive 2010/63/EU of the European Parliament and the Council on the protection of animals used for scientific purposes. Humane endpoints were predefined, and every effort was made to minimize animal suffering.

### 2.2. Biomaterial

The small intestine submucosa patches used in the study were purchased from Bejing Biosis Healing Biological Technology Co. (Bejing, China). Each patch measured 1.0 ± 0.2 cm in diameter, corresponding to the size of the created defect. Prior to implantation, patches were soaked in sterile 0.9% saline solution for 10 min to restore pliability.

### 2.3. Animals and Experimental Design

Fifteen adult male Wistar rats from the experimental station of our institution (Targu Mures, Romania) were included (median age of 1.5 years; median weight: 430 g; range: 344–480 g). Animals were housed individually in 2000 p Tecniplast cages purchased from Tecniplsast S.p.A (Buguggiate, Italy) under controlled laboratory conditions (22 ± 1 °C, 55 ± 10% relative humidity, 12 h light/12 h dark cycle) with free access to standard chow and water. Enrichment was provided in the form of nesting material. Food was withheld 24 h before surgery, while water remained available. Allocation to procedures was random. Sample size was chosen based on prior studies with similar models, aiming to balance animal use with sufficient power to detect histological differences. A control group without SIS graft application was not included in the experimental design. This decision was based on ethical considerations, to avoid leaving full-thickness gastrointestinal defects uncovered, which would have posed a high risk of suffering, and on pragmatic grounds, given the limited number of animals available. For a direct comparison between spontaneous healing and SIS-assisted repair, future studies may incorporate a control group with conventional anastomosis.

### 2.4. Anesthesia and Surgical Procedure

General anesthesia was induced with 4% isoflurane in oxygen (flow 2 L/min) and maintained at 1.5–2%. Depth of anesthesia was monitored by pedal withdrawal reflex and respiratory pattern. Animals were positioned on a heated pad to prevent hypothermia. Under sterile conditions, a midline laparotomy was performed. Three full-thickness defects were created: one on the anterior gastric wall, one on the antimesenteric side of the jejunum (5 cm from the Treitz ligament), and one on the descending colon. Full-thickness circular defects were standardized to 1.0 ± 0.1 cm in diameter using a sterile template.

Each defect was immediately covered with an SIS patch of equivalent size and fixed in place with 5-0 polypropylene interrupted sutures. The abdominal wall was closed with subcuticular sutures reinforced externally with a thin layer of cyanoacrylate adhesive.

### 2.5. Postoperative Care

Postoperatively, rats were observed until full recovery from anesthesia and then monitored daily by the research team. Analgesia was provided with meloxicam (1 mg/kg subcutaneously, every 12 h for 48 h), with additional doses administered if signs of pain were noted. Food and water intake, and general activity, were recorded daily. Oral feeding was resumed on the first postoperative day.

### 2.6. Second Surgical Intervention and Tissue Harvesting

At 21 days post-surgery, animals were euthanized according to Law No. 43/2014 of the Romanian Parliament and Directive 2010/63/EU of the European Parliament and the Council on the protection of animals used for scientific purposes. A second laparotomy was performed. The peritoneal cavity was examined for signs of intra-abdominal inflammation, peritonitis, abscesses, fibrinous coverings, fistulas, and adhesions. The anastomosis site was checked for signs of wound dehiscence, fistulas, and adhesions. Adhesions were graded according to the Zühlke classification: Grade 0 = none; Grade 1 = easily separable; Grade 2 = requiring blunt dissection; Grade 3 = requiring sharp dissection; Grade 4 = organ walls fused. For reporting simplicity, we categorized Grades 1–3 as light, fixed, and solid, respectively. Anastomotic sites together with adjacent healthy tissue were harvested, rinsed in saline, and fixed in 10% neutral buffered formalin for histological analysis.

### 2.7. Histological Analysis

Specimens were fixed for 48 h, embedded in paraffin, and sectioned at 4 µm. At least three non-consecutive sections from each grafted site were obtained. Sections were stained with hematoxylin and eosin (HE) and evaluated for epithelial coverage, fibrosis, neovascularization, and inflammatory cell infiltration. Fibrosis was graded as mild, moderate, or severe, and inflammatory infiltrates were categorized as mononuclear or mixed. Histological samples were anonymized and assessed by two independent observers blinded to segment location.

## 3. Results

**Figure 1:**
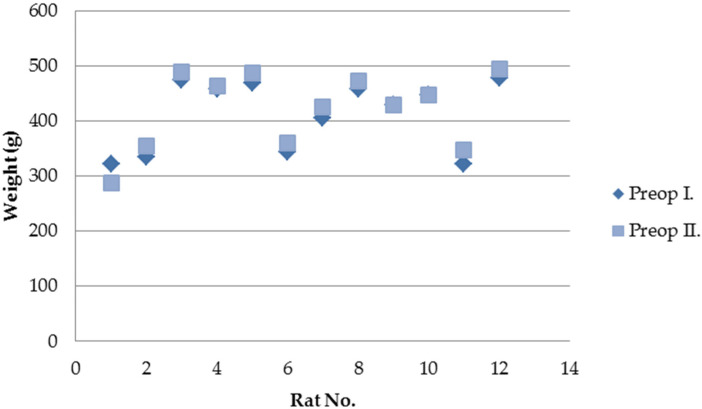
Weight changes in surviving rats between the two surgical interventions.

Of the 15 rats operated on, three died during the first postoperative week. Two animals showed no identifiable intra-abdominal pathology at necropsy, while one presented SIS patch displacement with minimal leakage. The remaining 12 animals survived until the second surgical intervention. All survivors tolerated oral feeding from the first postoperative day, and 11 gained weight during the follow-up period (Figure 1). None of the surviving rats developed clinical signs of illness.

### 3.1. Macroscopic Examination

**Figure 2:**
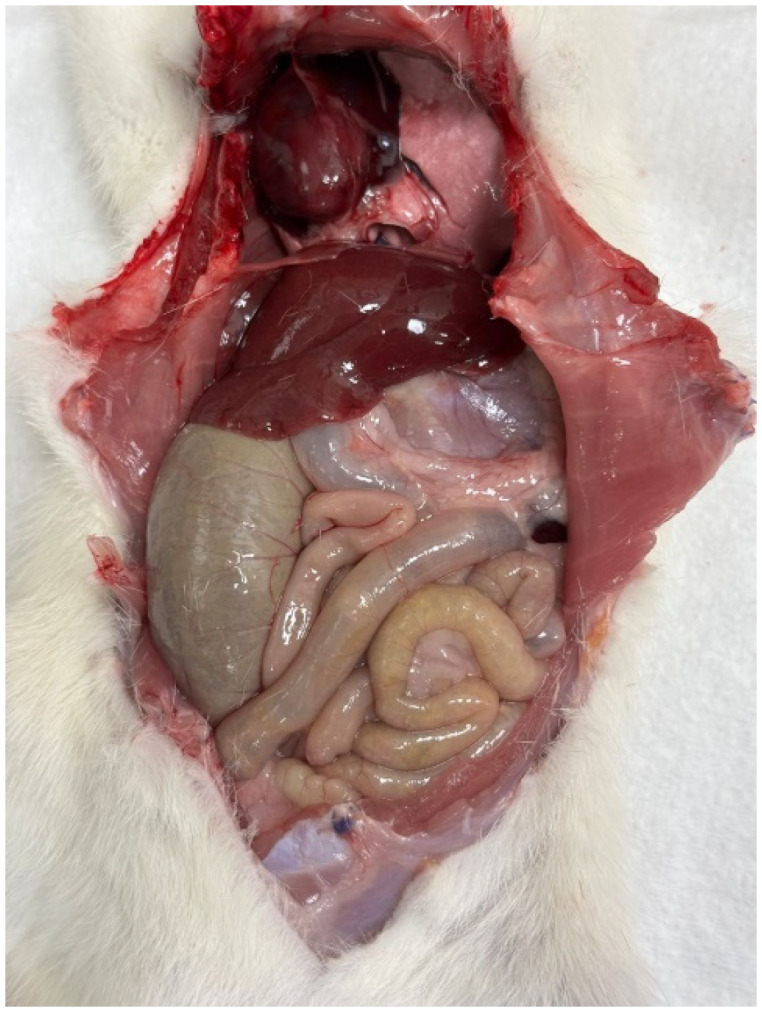
Without visible adhesion.

**Figure 3:**
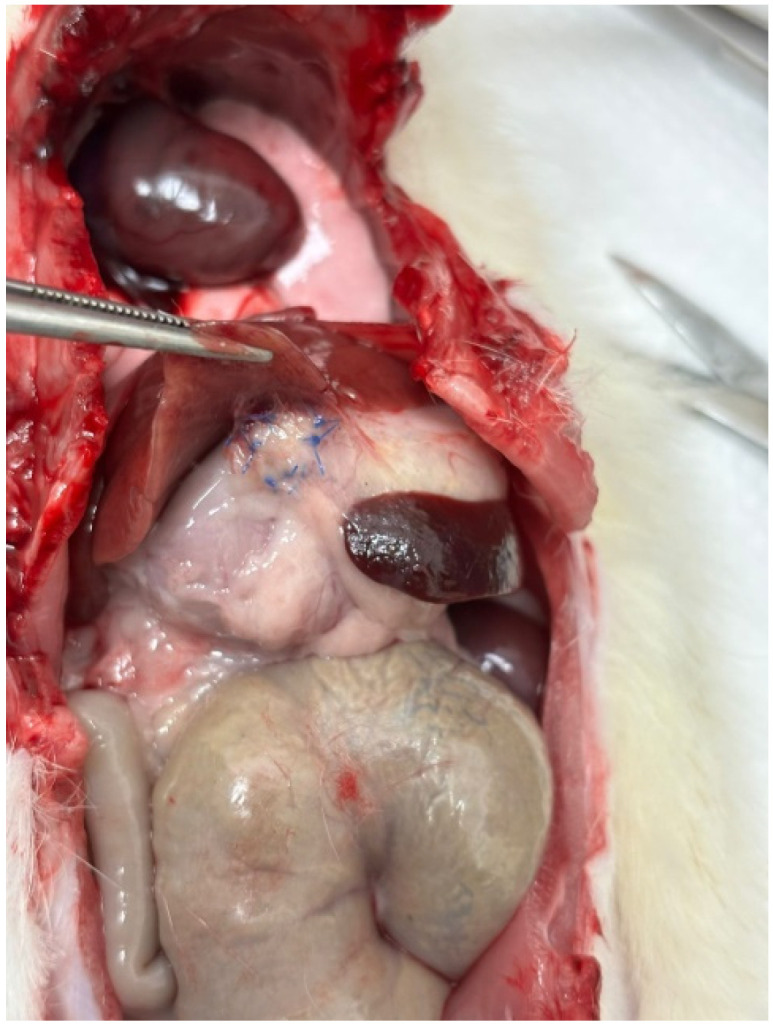
Stomach–liver adhesion.

Macroscopic examination of the abdominal cavity after the second surgery (Figure 2) showed no signs of stercoral peritonitis, intra-abdominal abscess, fistulas, leakages, stenosis, or bowel wall necrosis in any of the cases (Table 1). Macroscopic detection of the patches was difficult due to the coverage of the patched area with adhesions, but it was possible by recognizing the non-absorbable sutures. Adhesions were common, detected in 11 of 12 animals, and were generally mild to moderate. Adhesions most frequently involved the stomach and liver (4/12) (Figure 3), interileal loops (3/12), ileum and spleen (2/12), ileocolic region (1/12), and ileum and omentum (1/12).

### 3.2. Microscopic Examination

**Figure 4:**
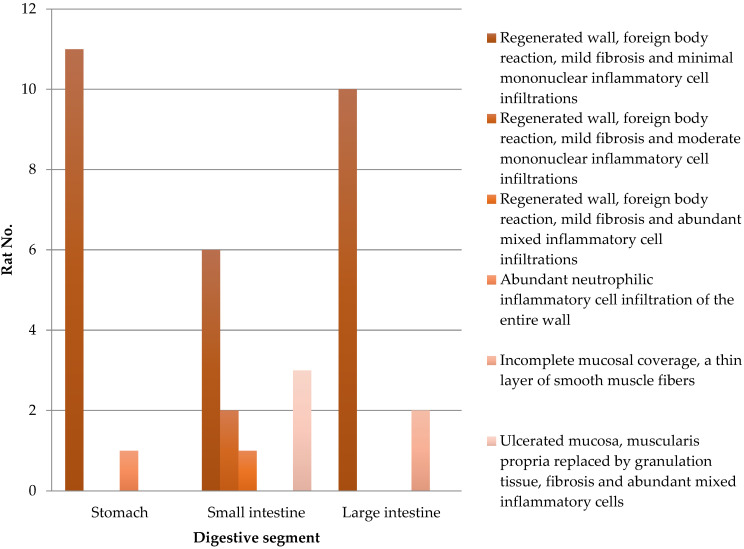
Histological analysis of the stomach and small and large intestines from 12 rats after SIS matrix integration.

Every implanted SIS graft is integrated into the gastrointestinal wall. The results of the histological analysis are detailed in Table 2 and Figure 4. Complete mucosal coverage was seen in 12 stomach, 9 small intestine, and 10 large intestine specimens. Granulation tissue and mixed inflammatory cell accumulations replaced the submucosa in three cases of small intestine segments. Incomplete mucosal coverage was seen in two large intestine fragments. Early regeneration with prominent vascular proliferation of the mucosa was seen at the edge of the former defect. Foreign-body giant cell reaction was limited to the areas of the suture.

**Figure 5:**
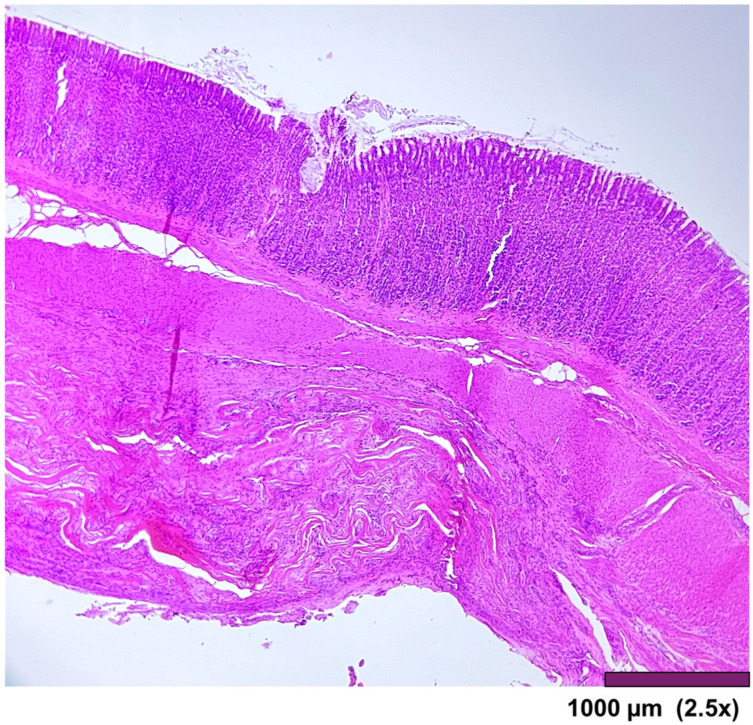
All layers in the stomach wall-mucosa, submucosa, muscularis propria, and serosa. There is a minimal architectural difference between the native and the regenerated stomach, with mild fibrosis and moderate infiltration of inflammatory cells in the subserosal layer. (2.5×, HE).

**Figure 6:**
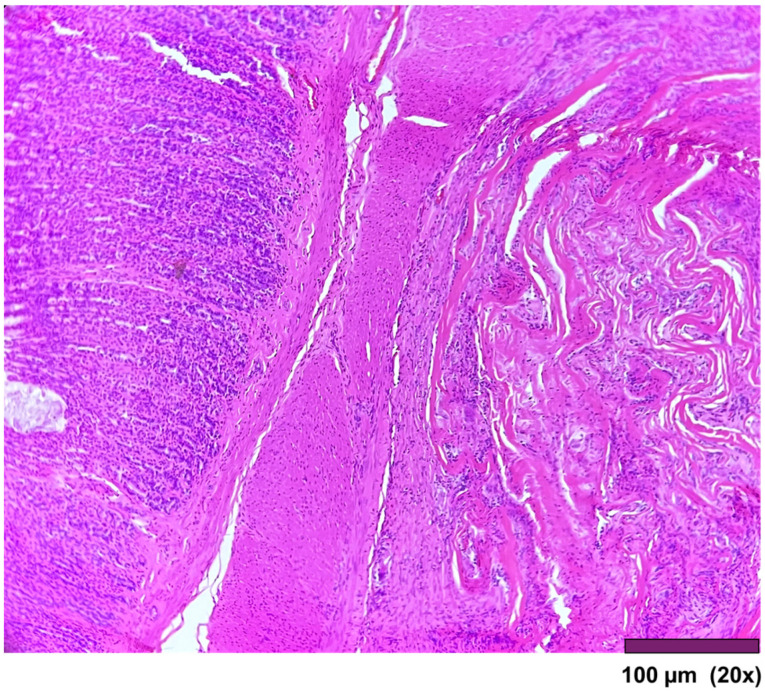
Stomach: Muscularis propria was replaced by a thick layer of fibrosis (20×, HE).

The stomach wall showed (Figure 5) minimal architectural difference between the native and the regenerated stomach fragment, with mild fibrosis and moderate infiltration of inflammatory cells in the subserosal layer. In one sample, the SIS-covered stomach portion of the muscularis propria was replaced by a thick layer of fibrosis (Figure 6).

**Figure 7:**
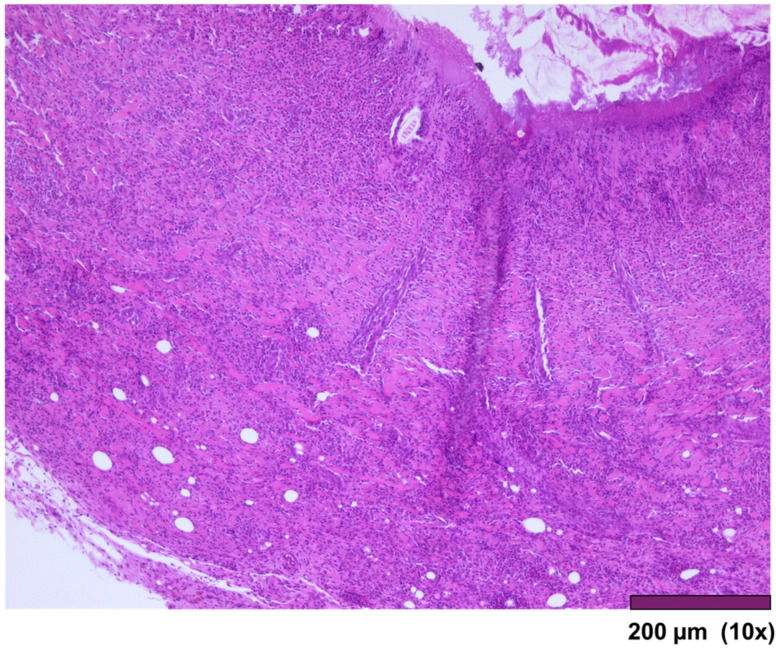
Small intestinal wall covered by granulation tissue and early fibrosis, both with neovascularization and infiltration of inflammatory cells (10×, HE).

**Figure 8:**
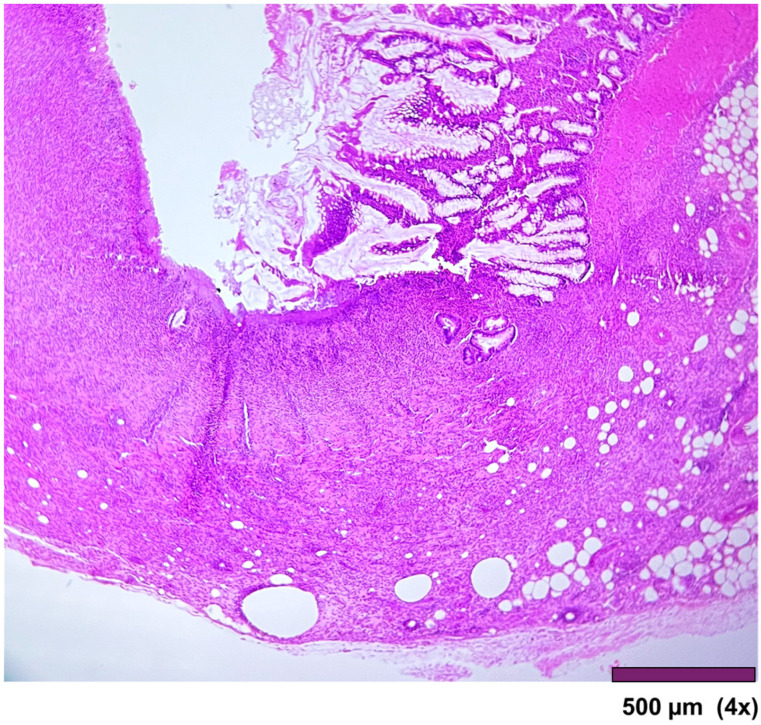
Small intestine with ulcerated mucosa and muscularis propria replaced with granulation tissue, fibrosis, and inflammatory cells (4×, HE).

The small intestine wall regenerated with fibrosis and with different degrees of mononuclear inflammatory cell infiltrations. Neovascularization could be seen as well (Figure 7). In three cases, ulceration of the mucosa was observed, where the muscularis propria was replaced by granulation tissue and inflammatory cells (Figure 8).

**Figure 9:**
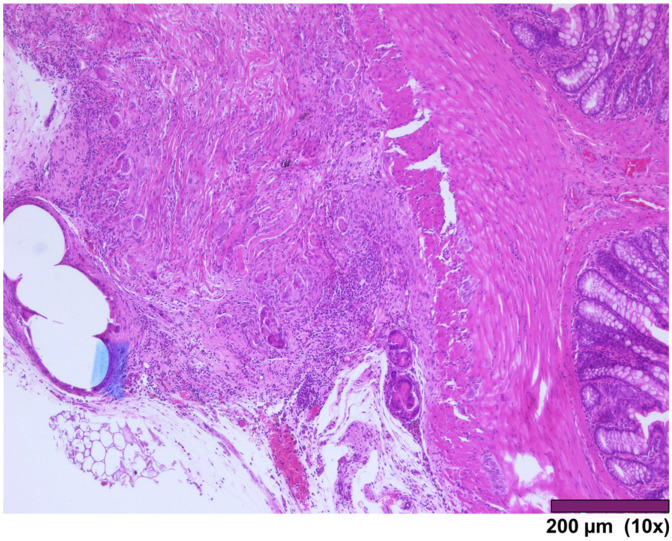
Large intestine: Completely regenerated mucosa, lamina propria, submucosa, muscularis propria, subserosal fat, and granulation tissue with chronic inflammation and foreign body reaction (10×, HE).

**Figure 10:**
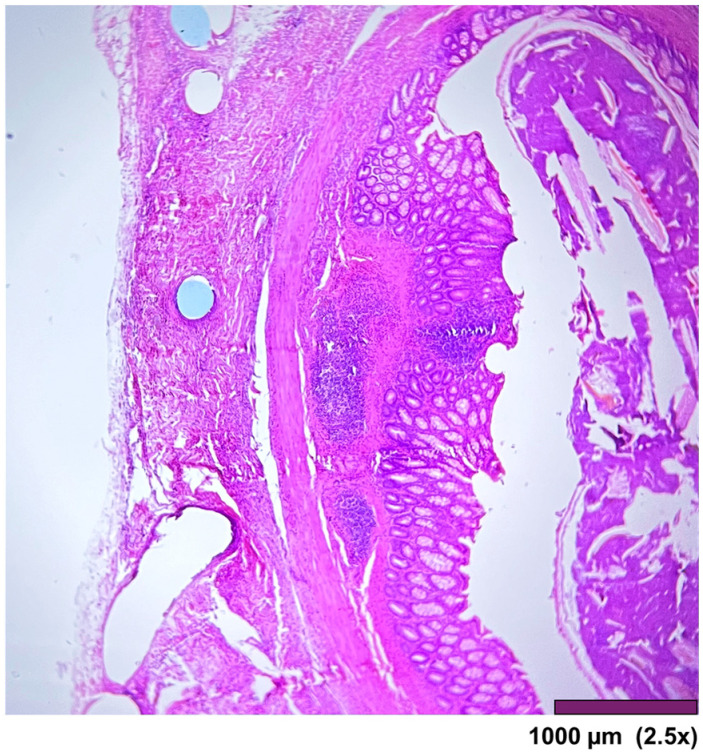
Large intestine: mucosa—top layer, submucosa— between mucosa and muscle layer. The mucosal layer completely covers the anastomosis. The holes in the middle are artifacts due to the removal of the anastomotic sutures (2.5×, HE).

The large intestine wall regenerated completely after surgery, as seen in Figure 9 and Figure 10.

## 4. Discussion

### 4.1. Biomaterial Types and Properties

Biomaterials can be broadly classified into three categories: naturally derived (collagen, alginate), acellular (bladder mucosa or small intestinal submucosa), and synthetic polymers (polylactic-coglycolic acid, polyglycolic acid, and polylactic acid) [18]. Extracellular biomaterials are collagen-based matrices that are nonimmunogenic and promote host responses such as tissue regeneration, neovascularization, and site-specific restoration of tissue structure [19].

The most common, experimentally and clinically studied extracellular matrix is derived from porcine small intestinal submucosa. Badylak et al. first described its preparation method, which has since become the reference standard in tissue engineering and regenerative medicine [20]. SIS is commercially available and already in clinical use for the treatment of anal fistula and hernia repair [21,22].

### 4.2. Regeneration Mechanisms of SIS

The regenerative process in SIS-patched areas involves angiogenesis, cell migration, and differentiation. SIS predominantly consists of collagen types I, III, and IV, proteoglycans, glycosaminoglycans, and glycoproteins that support angiogenesis. Cellular migration and attachment-responsible molecules (fibronectin and heparin sulfate proteoglycan), growth factors (fibroblast growth factor—FGF, vascular endothelial growth factor—VEGF, and transforming growth factor (TGF)) have been identified in SIS composition after sterilization procedures [19].

Experimental studies support this regenerative capacity. Demirbilek et al. observed fibrovascular healing with mononuclear and fibroblastic invasion in rabbits, followed by columnar epithelium at 4 weeks and villus-like architecture with goblet cells at 6 weeks [23]. Our results are consistent with these reports, showing early epithelial recovery, fibrosis, inflammatory infiltration, and neovascularization within 3 weeks of SIS implantation.

### 4.3. SIS Limitations

Not all studies demonstrate complete mucosal transformation. Lee et al. reported a lack of mucosal changeover in an isolated intestinal segment with an interposed SIS graft in rats, suggesting that regeneration may result from SIS contraction rather than true intestinal tissue transformation [24]. The contraction rate of the SIS could be species-dependent. Hoeppner et al. described SIS shrinkage leading to bowel obstruction [25]. In our study, patch shrinkage was visible macroscopically but did not cause stenosis.

A frequent complication after intestinal surgery was adhesion formation, which was observed in most cases. However, adhesions were not associated with abscesses, necrosis, or leakage, suggesting that they were part of the normal healing and SIS integration process. The presence of transmural inflammation in some small intestinal samples raises concerns, but we interpret these changes as part of the early regenerative phase, when fibrosis, granulation tissue, and inflammatory infiltration are expected to coexist.

### 4.4. Animal Model Choice

Longevity in male Wistar rats is 1.7–3.2 years in laboratory settings [26]. At one year, a Wistar rat is considered a mature adult with early immunosenescence and both central and peripheral lymphoid compartment remodeling [27].

We have chosen rats of a median age of one year to better approximate compromised clinical conditions, similar to preterm infants with NEC, in whom metabolic derangements and hemodynamic instability’s consequences are risk factors for poor anastomosis healing. To our knowledge, this is the first study evaluating SIS patch integration in the stomach, small intestine, and large intestine within one animal model.

### 4.5. Comparison with Previous Studies

De la Fuente et al. reported gastric wall regeneration after a two-layered SIS patch, where granulation tissue replaced all gastric wall layers at 3 weeks [11]. Ueno et al. found smooth muscle proliferation internally and fibrosis externally in the regenerated stomach wall [28]. In contrast, we found minimal architectural differences. Our one-layer SIS patch produced only mild fibrosis and moderate inflammatory infiltration in the subserosal layer, with one case showing diffuse neutrophil infiltration in the entire wall.

In the small intestine, Wang et al. evaluated the morphologic regeneration in rats after using a tubular SIS graft in the middle of an ileal Thiry–Vella loop, which was used to construct an ileostomy. They described that the mucosal epithelial layer started to cover the luminal surface with evident neovascularization [29,30]. Consistently, we observed early mucosal recovery with a foreign-body reaction limited to the areas of suture, mild fibrosis, and minimal mononuclear inflammatory cell infiltrations. In two cases, the muscularis propria was replaced by granulation tissue, fibrosis, and abundant mixed inflammatory cells.

In the large intestine, Hoeppner et al., 30 days post-implantation, found granulation tissue with neovascularization but limited mucosal regeneration, along with diffuse inflammation of the wall, foreign body granuloma, lymphocytes, and macrophage infiltration without abscess or infection [25,31,32]. In our study, 10 cases showed regenerated walls, foreign body reaction limited to the areas of suture, mild fibrosis, and minimal mononuclear inflammatory cell infiltrations. In two cases, there was incomplete mucosal coverage with a thin layer of smooth muscle fibers.

### 4.6. Strengths of the Study

This work has a few notable strengths. The model itself is one of them: by repairing defects in the stomach, small intestine, and colon at the same time, we tried to reproduce the multifocal and length-preserving challenges we face in necrotizing enterocolitis and other pediatric conditions. Despite the severity of this setup, most animals survived, and none developed leaks, peritonitis, or stenosis, which suggests that SIS can be applied safely across different parts of the gastrointestinal tract. Another strength is the histological evidence of regeneration. We found complete mucosal coverage in all gastric samples and in the majority of small-bowel and colonic sites, usually accompanied by only mild fibrosis and minimal inflammation. The foreign-body reaction was limited to the sutures rather than the patch itself. Adhesions were common but mostly mild to moderate and did not cause secondary problems, which supports the overall tolerance of the material. Finally, we used standardized defects and a blinded review of the histology, which adds confidence to the consistency of our findings. Taken together, these points provide a solid starting place for moving toward longer follow-up and, ultimately, into larger animal models.

### 4.7. Limitations of the Study

There are also some clear limitations to our work. We did not include a control group, such as a group for secondary healing, primary closure, or resection with anastomosis. Our primary goal was to establish the feasibility of simultaneous SIS repair at multiple gastrointestinal sites. Comparative studies are planned to determine whether SIS confers advantages over standard surgical techniques. The 3-week endpoint represents an early healing window. Longer-term studies with 8–12 weeks of follow-up will be required to assess full remodeling. Finally, because the study was not powered for inferential statistics, the findings should be interpreted as descriptive proof-of-concept data rather than definitive comparative outcomes.

### 4.8. Future Perspectives

Although several studies suggested that SIS could be used as a scaffold in gastrointestinal regeneration, functional aspects of the neointestine remain insufficiently studied. Peristaltic activity and absorptive mucosal function need to be evaluated in long-term models. Our findings should be interpreted as preliminary. They represent an early stage of healing rather than full restitution of normal architecture. While HE provided valuable insights into mucosal coverage, fibrosis, and inflammatory responses, it does not allow precise characterization of inflammatory subsets, proliferative activity, or angiogenesis. Immunohistochemical markers such as CD3, CD20, CD68, Ki67, or VEGF would offer a more objective understanding of regenerative processes and will be incorporated into future experiments. Future experiments should combine immunohistochemistry and functional assays to strengthen the evidence.

Translation of these results into neonatal practice faces challenges. The small size and fragile intestines of neonates, the immaturity of their immune system, and the frequent exposure to a septic peritoneal environment in NEC may all influence graft integration. Larger animal models, such as newborn piglets, could provide a more relevant approximation of neonatal physiology. These models would allow testing of mechanical resistance under peristalsis, characterization of local and systemic immune responses, and long-term evaluation of tissue regeneration under septic conditions. Further confirmation in translational models could lead to clinical trials, in which SIS could become a valuable alternative in the surgical management of NEC, helping to preserve bowel length and reduce the need for extensive resections.

## 5. Conclusions

In this experimental model, SIS patches integrated successfully into full-thickness gastric, small intestinal, and colonic defects. After three weeks, most grafted sites showed complete mucosal coverage, with fibrosis, inflammatory infiltrates, and neovascularization consistent with an early regenerative phase.

This is, to our knowledge, the first study to evaluate SIS simultaneously in three gastrointestinal segments within one animal model, simulating the multifocal injuries seen in conditions such as necrotizing enterocolitis. While the exclusive use of HE staining and the short follow-up period limit the depth of interpretation, our results provide preliminary evidence that SIS can promote regeneration across different regions of the gastrointestinal tract.

Further studies with larger sample sizes, extended follow-up, and immunohistochemical and functional analyses are needed to clarify the long-term quality of regeneration. Translational studies in neonatal-relevant large animal models, such as piglets, will be essential to determine whether these encouraging results can be reproduced under conditions that approximate the clinical setting. Ultimately, if confirmed, SIS may evolve into a valuable alternative in the surgical management of NEC, offering the potential to preserve bowel length and improve outcomes.

## Figures and Tables

**Table 1:** Macroscopic examination of the anastomosis.

	Number (*n* = 12)
Signs of wall necrosis	0/12
Affected segment stenosis	0/12
Anastomotic leakage/ulcer	0/12
Adhesions—localization	
Stomach–liver	4
Interileal	3
Ileum–spleen	2
Ileocolic	1
Ileum–omentum	1
Adhesions–quality rate	
Light adhesions	6/12
Fixed adhesions	4/12
Solid adhesions (removable only with damage)	1/12

**Table 2:** Histological analysis of the stomach and small and large intestines from 12 rats after SIS matrix integration.

	Stomach	Small Intestine	Large Intestine
Regenerated wall, foreign body reaction, mild fibrosis, and *minimal * mononuclear inflammatory cell infiltrations	11	6	10
Regenerated wall, foreign body reaction, mild fibrosis, and *moderate* mononuclear inflammatory cell infiltrations	0	2	0
Regenerated wall, foreign body reaction, mild fibrosis, and *significant* mixed inflammatory cell infiltrations	0	1	0
Neutrophil inflammatory cell infiltration of the entire wall	1	0	0
Incomplete mucosal coverage, with a thin layer of smooth muscle fibers	0	0	2
Ulcerated mucosa, muscularis propria replaced by granulation tissue, fibrosis, and abundant mixed inflammatory cells	0	3	0

## Data Availability

Data is contained within the article. The original contributions presented in this study are included in the article. Further inquiries can be directed to the corresponding author.

## Abbreviations

The following abbreviations are used in this manuscript:

NEC	Necrotizing enterocolitis
SIS	Small intestine submucosa
HE	Hematoxylin and eosin
